# A Pilot Dance Intervention to Encourage Physical Activity Engagement for Adolescent Girls with Intellectual Disabilities

**DOI:** 10.3390/ijerph19084661

**Published:** 2022-04-12

**Authors:** Aviva Must, Linda G. Bandini, Carol Curtin, Katherine M. Rancaño, Misha Eliasziw, DJ Tybor, Heidi Stanish

**Affiliations:** Department of Public Health and Community Medicine, Tufts University School of Medicine, 136 Harrison Avenue, Boston, MA 02111, USA; linda.bandini@umassmed.edu (L.G.B.); carol.curtin@umassmed.edu (C.C.); katherine.rancano@tufts.edu (K.M.R.); misha.eliasziw@tufts.edu (M.E.); david.tybor@tufts.edu (D.J.T.); heidi.stanish@umassmed.edu (H.S.)

**Keywords:** dance, intellectual disabilities, physical activity, enjoyment, engagement, adolescent girls

## Abstract

Adolescent girls with intellectual disabilities (ID) are at risk for low physical activity (PA) participation due to their limited opportunities. Purpose: To evaluate the feasibility and preliminary efficacy of a 12-week dance intervention to promote engagement in moderate-to-vigorous PA (MVPA) and increase cardiorespiratory fitness. Methods: The 12-week intervention included two 75-min weekly dance sessions. Continuous heart-rate (HR) monitoring assessed time spent below/at/above each girl’s target HR zone. Cardiorespiratory fitness was measured by the 6-min walk test (6MWT). Survey items assessed participant enjoyment and participant and parent satisfaction. Results: The mean (SD) age of 18 adolescent girls was 17.3 (2.7) years. Overall, girls attended 88% of sessions and spent 52.3% of each session in MVPA. Mean MVPA was unchanged across the 12 weeks, but the pattern differed across the three sites. We observed a non-significant pre-post increase of 74.6 feet on the 6MWT. Post-intervention surveys indicated that most girls liked the program, perceived improved fitness, and wished to continue dancing. The majority also reported a preference for a girls-only dance program exclusively for those with ID. Conclusion: Our findings suggest that dance is viable for promoting PA for girls with ID. More frequent exercise training is likely needed to improve cardiorespiratory fitness.

## 1. Introduction

Intellectual disability (ID) is a developmental disability characterized by significant limitations in both intellectual functioning and adaptive behavior and is present before 22 years of age [1]. Intellectual functioning includes problem solving, learning, and reasoning (i.e., general mental capacity) and adaptive behavior refers to a collection of social, practical, and conceptual skills that are used in everyday life (e.g., language, occupational skills, number concepts, healthcare, self-esteem) [1]. Recent estimates indicate that ID affects approximately 3.2% of the global population of children and adolescents [2], with an estimated prevalence for children in the United States of 1.2% [3,4]. The five most common types of ID are autism spectrum disorder, Down syndrome, fragile X syndrome, and fetal alcohol syndrome [4]. ID is a lifelong disability and is associated with persistent functional limitations that often necessitate support in a variety of settings, contexts, and activities.

Participation in regular physical activity has many health benefits for children and adolescents, including increased cardiorespiratory fitness, healthy weight, increased bone mass, and fewer symptoms of anxiety and/or depression [5,6]. Yet, evidence indicates that youth with ID have low levels of physical activity [7,8,9,10,11]. Globally, adherence to physical activity guidelines among youth with ID is low, though the reported range is wide: from 0% of children with ID meeting guidelines [12,13] to 24% [14]. Consequentially, children and adolescents with ID have poor health-related fitness [15,16,17,18] and a high prevalence of obesity [19,20,21,22]; they also experience health inequities compared to youth with typical development (TD) [23]. The World Health Organization’s (WHO) Global Action Plan on Physical Activity 2018–2030 [24] acknowledges the disparities in physical activity among individuals with disabilities. For the first time, the 2020 WHO guidelines on physical activity and sedentary behavior are inclusive of children and adolescents living with a disability and make specific recommendations for this population subgroup [25,26].

A multitude of individual/personal, family, social, and environmental factors hinder opportunities for youth with ID to be physically active. Examples of these include low skills, lack of accessible programming, and low motivation [27,28]. Limited evidence supports gender as a correlate of low physical activity participation in youth with ID, with girls being less active than boys [13,29]. Such gender-based disparities are well-documented in the general population [30,31]. In addition, physical activity levels decline from childhood through adolescence in both youth with ID [29,32] and youth with TD [6,33]. Children with ID—especially adolescent girls—need feasible and effective interventions to increase their physical activity engagement.

Dance is a form of physical activity that stands out as enjoyable and popular among adolescent girls [34,35,36,37]. Dance programs improve a host of mental and physical health outcomes in young people [38,39,40,41,42]. Dance increases social interaction, camaraderie, personal expression, intrinsic motivation, and creativity [43] in all youth, regardless of ability [44,45]. In our observational study of teenagers with ID, 75% of adolescent girls ranked dance (mostly at home) as the most common leisure time physical activity [10]. Dance has physical, social, and psychosocial benefits for children with various disabilities including ID [46]. Dance intervention studies have targeted subgroups of girls, such as racial/ethnic minorities [41,42,47] and low-income populations [48], but to our knowledge, none have determined the ability of dance programs to help adolescent girls with ID obtain the recommended 60 min of moderate to vigorous physical activity (MVPA) daily [49].

Despite the compelling reasons to believe that dance represents a suitable approach to increasing physical activity in community settings for girls with ID, there is a dearth of research in this area, perhaps due to the difficulty of developing programs that appeal to the interest, motivation, and learning styles of adolescent girls with ID. We postulated that a structured, supportive, and socially engaging dance program would promote MVPA among adolescent girls with ID and increase their cardiorespiratory fitness. Therefore, we aimed to determine the feasibility of a 12-week dance program for engaging adolescent girls with ID in MVPA and to assess the preliminary efficacy of the program to improve cardiorespiratory fitness. The overall goal of this line of research is to build evidence that supports intervention efforts to reduce the physical activity disparities experienced by individuals with ID.

## 2. Materials and Methods

### 2.1. Participants

We recruited girls with ID in the greater Boston area, who were 13 to 21 years of age, to participate in a 12-week dance program through public school special education programs, parent advisory councils, and local disability organizations. According to the US Individuals with Disabilities Education Act, youth with disabilities have access to educational services until their 22nd birthday, and so may remain in school until age 22 [50]. We determined eligibility and gave a study overview during parent/guardian telephone interviews. Eligible participants had an IQ score ≤75 as confirmed by parent report, were age 13–21 and were able to verbally communicate, follow simple instructions, and provide assent/consent in English. We excluded participants with untreated cardiac conditions, seizure disorder, psychiatric conditions (e.g., schizophrenia, bipolar disorder, or psychosis), heart disease, cancer, physical disabilities, orthopedic injury/impairment, disruptive behaviors (e.g., aggression, property destruction, or frequently running away from a program area) and any participants engaging in aerobic exercise outside of school for more than 60 min/day.

An in-person enrollment visit was scheduled for participants who met the screening criteria. At enrollment, eligibility was confirmed, the study protocol was thoroughly reviewed, parents provided informed written consent, and participants indicated their willingness to participate by signing an assent or consent form (depending on their age and guardianship status) that was read aloud to them. Written approval from a primary care physician was also required to participate. All procedures were approved by the university’s review board for the protection of human subjects.

### 2.2. Settings

The 12-week intervention was conducted at three sites in eastern Massachusetts in order to recruit an adequate sample of girls with ID. Although each site varied by location/setting, number of participants and schedule (i.e., days/times), the components of the intervention were the same at all three sites, including the dance instructor and all study procedures. At Site A, the dance program was conducted during in-school hours at a fitness studio within a YMCA (an international community-based youth organization with branches across the US) located in an urban neighborhood of a small city. The special education program of the local public school transported the participating students to and from the YMCA so they could participate in the dance program during regular school hours. The Site B program was conducted during out-of-school time at a fitness studio within another YMCA fitness studio located in a suburban neighborhood in a second small city. Participants were transported to and from the facility by a parent, guardian, or other family members. At Site C, the dance program sessions were delivered during school hours in the gymnasium of a public high school located in a densely populated section of a large city. This school has a large special education program and participation in the dance program for Site C was restricted to female students with ID who were currently enrolled in the school. At Sites A and C, a teacher accompanied the girls to the facility/gymnasium, but they were not involved in the dance program and were not present during the dance sessions or testing. In-person contact with parents at these sites was limited to the screening and enrollment process, whereas at Site B there was ongoing in-person interaction with parents at drop-off and pick-up at the YMCA. All three sites completed the study within a single year.

### 2.3. Intervention

Study Design. The pilot intervention used a single-arm trial design for cardiorespiratory fitness. Engagement and satisfaction measures were assessed at baseline and then weekly post-intervention.

Group Dance Sessions. Group dance sessions were conducted twice weekly for 75 min over the 12-week intervention period. Sessions were led by a trained dance instructor with experience teaching children and youth in local dance programs. A student research assistant was present to assist the instructor and support participants. The first 10 min of each session was used to greet participants, provide an overview of the content for the class, and provide an opportunity for participants to socialize. The remainder of the session included: a 10-min warm-up period, 30–45 min of dance practice/choreography, and 5–10 min of cool-down activities.

Two dance styles were included in the 12-week program: 6 weeks of hip hop followed by 6 weeks of jazz. Dance sessions included simple choreography of each style (i.e., moves and routines) designed to be easy to learn and master by girls with ID without extended periods of instructional time when participants would be inactive. The dance moves and routines were also intended to be of moderate-to-vigorous intensity and the instructor-led activities were designed to keep participants moving at a level that would elicit their target HR. The target HR zone for participants during the dance was established as 60–79% of their age-predicted maximum HR (220 beats/min—age) [51]. Participants wore a Polar^®^ HR monitor during each dance session to continuously monitor the intensity of their engagement and to provide them with feedback. Participants were taught to read the output on the HR watch and to recognize their own target HR zone. There were frequent HR checks throughout the dance sessions. During each session, participant engagement in MVPA by Polar^®^ HR monitors (Polar Electro, Kempele, Finland) is reflected by the percentage of time spent in or above the participant’s target HR zone.

At-home Dance Practice. Participants were instructed to dance at home at least twice each week for 45–60 min to increase their frequency of physical activity participation and to practice routines taught during the group sessions. Participants were encouraged to dance freely for the at-home practice or to use instructional videos that were provided and illustrated each dance style to support and motivate participants’ engagement. The videos featured the dance instructor leading routines taught in class with demonstration, verbal instruction, and encouragement. The video format was similar to the group dance classes, with 10 min of warm-up, 30–45 min of dance practice, and 5 min of cool-down. In order to monitor the intensity of their activity during at-home dance and stay in their target HR zone, participants were asked to wear a Fitbit^®^ Charge HR™ activity monitor (Fitbit, San Francisco, CA, USA) that was provided at the enrollment visit. Participants were encouraged to schedule consistent days/times to dance at home so that it became part of their weekly routine.

### 2.4. Feasibility Measures

Attendance and Retention. Attendance at each group dance session was recorded and the number of participants who withdrew from the study prior to week 12 was documented.

Engagement during Group Dance Sessions. Continuous HR monitoring was used to estimate the level of engagement in MVPA during each dance session using Polar^®^ monitors. Participants put on a monitor (chest strap and output watch) at the start of each session and were asked to wear it for the full duration of the session. Assistance to put on the monitor and ensure proper placement was provided by a female member of the research team in a private space. HR data were stored and then downloaded using the Polar^®^ software.

Participation in At-home Dance Practice. Participation in at-home dance practice sessions was estimated using the Fitbit^®^ Charge HR™ activity monitor and an activity log. The device displays, records, and stores HR data and was synched wirelessly to a computer, tablet, or smartphone. Data were then retrieved from each participant’s Fitbit^®^ dashboard throughout the program. Participants, with help from a parent as needed, were instructed to manually record the date, start time, and end time of each at-home practice session on an activity log that was provided. The log was designed to assist in verifying the information collected from the Fitbit^®^ Charge HR™, particularly the time period that the participant danced, and was a secondary source of documenting participation at home. Participants were instructed to bring the completed log to the group dance session each week.

Enjoyment and Satisfaction. A survey was used to assess participant enjoyment of and satisfaction with the dance program. The 5-item survey was administered following the second dance session each week for weeks 3–12. Four questions asked about the enjoyment of the weekly dance style, music, at-home practice, and dancing with other participants, and one question asked participants about their level of effort when dancing that week. The survey was administered verbally to participants and response choices were recorded by a member of the research team. Three response options (i.e., “liked it”, “it was ok”, and “did not like it”) were presented and each option had a happy, neutral, or sad face to offer participants a visual cue when selecting a response. Few participants reported that they “did not like” components of the program; therefore, responses of “did not like” were combined with “just okay” and binary variables were created to indicate participants that “liked” components of the program. To examine participant enjoyment of at-home videos, a binary variable was created to indicate participants that tried and liked the at-home videos (i.e., 0 represents participants that did not try the at-home videos and participants that tried them but did not like them).

Surveys to assess participant satisfaction with the dance program overall were administered at the end of the session at weeks 6 and 12. The participant survey included 16 questions that asked about perceived benefits of the dance program (e.g., fitness, learned new dance routines), satisfaction with the instructor, the HR monitors, and videos for at-home practice, interest in continuing to dance, and preferences for including girls not in special education (i.e., those without ID) and boys in future dance programs. The survey was administered verbally to participants and response choices were recorded. The parent satisfaction survey, administered at weeks 6 and 12, included 20 questions about key program elements, such as intensity and difficulty of dance activities, appropriateness of routines, instructional strategies, and support for at-home sessions, perceived benefits of the program for their daughter (e.g., fitness, physical activity), satisfaction with program length and schedule, and barriers and facilitators of participation. Most questions were Yes/No or had up to five response options, the survey included one open-ended question on suggestions for improving the dance program. Paper copies of the parent surveys were distributed and returned by mail. At Site B, some parents returned their surveys directly to study staff when transporting their children to the YMCA.

### 2.5. Outcome Measure

Cardiorespiratory Fitness. The 6-min walk test (6MWT) was used to measure cardiorespiratory fitness at baseline and after the 12-week dance program (within 5 days of the last group dance session). The test, which is widely used in research involving people with ID [52,53], was administered at the site of the dance sessions (i.e., YMCA or school gymnasium). Following a demonstration and simple verbal instructions, participants were instructed to walk as quickly as possible between two cones on a flat indoor surface for 6 min. To assist with pacing and motivation during the 6MWT, a research assistant walked slightly ahead of each participant and provided verbal encouragement.

### 2.6. Statistical Analyses

Participant characteristics were summarized using means for continuous and percentages for categorical variables. The average rates of session time spent in or above the participants’ target HR zone over the 12-week intervention were estimated from Poisson regression models with a compound symmetry covariance structure to account for participants’ repeated measures of HR over time. In the models, total time spent in or above the target HR zone was the outcome variable and total session time was the offset variable. Results are summarized as the percentage of session time spent in or above the target HR zone with corresponding 95% confidence intervals (CI) for all sites and each site separately.

Changes in the average distance walked in the 6MWT between pre- and post-intervention were assessed using linear mixed models. The models included a random intercept to account for the within-subject correlation. The fixed effects included site, timepoint (i.e., pre- and post-intervention), and a site-by-timepoint cross-product term. Results are summarized as the mean difference in distance walked with corresponding 95% CIs for all sites and each site separately. Despite four participants having missing data for the 6MWT at pre- or post-intervention, and not being included in this analysis, the baseline characteristics were similar between the analyzed sample and participants with complete 6MWT.

Participant attendance, satisfaction with program components, and activity logs returned for at-home use of the Fitbit^®^ Charge HR™ from week 3 to week 12 were estimated with logistic regression models using a generalized estimating equation (GEE) approach. A compound symmetry covariance structure was used in the models to account for the repeated measures from each participant. The results are summarized as average percentages with corresponding 95% CIs.

Participants’ satisfaction with the program post-intervention is summarized in terms of percentages. One participant from site B was missing post-intervention assessments and was excluded from this analysis. Parent satisfaction and parent perceptions of at-home videos post-intervention are summarized in terms of percentages and are limited to site B parents. Parents from site A and site C were not included because they had limited knowledge of the intervention’s content and delivery, given that the intervention at these sites was carried out during the school day. SAS version 9.4 was used for all statistical analyses and results with *p*-values less than 0.05 are considered to be statistically significant.

## 3. Results

Eighteen adolescent girls with ID participated in the intervention at the three sites of the 12-week dance program. Table 1 displays demographic characteristics overall and by study site. Overall, the mean (SD) age of the sample was 17.3 (2.7) years. The sample was diverse, with the greatest diversity in Site C. On average, slightly fewer than half of the girls’ parents had earned less than a bachelor’s degree, with striking differences by site: all of the Site B participants had at least one parent with a college education or more, whereas those participating in Site A had no parents with a college education or more.

**Figure 1:**
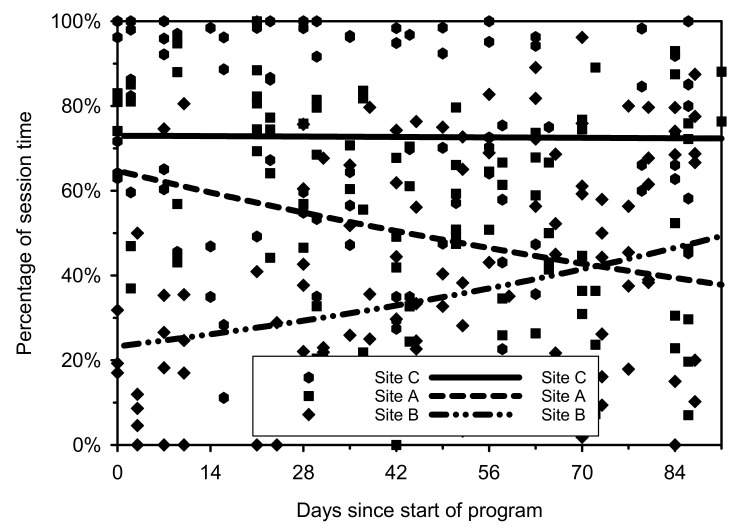
Scattergram of percentage of session time spent in or above participant target heart rate zone across time by site. The fitted regression lines are derived from a multivariable Poisson model.

**Figure 2:**
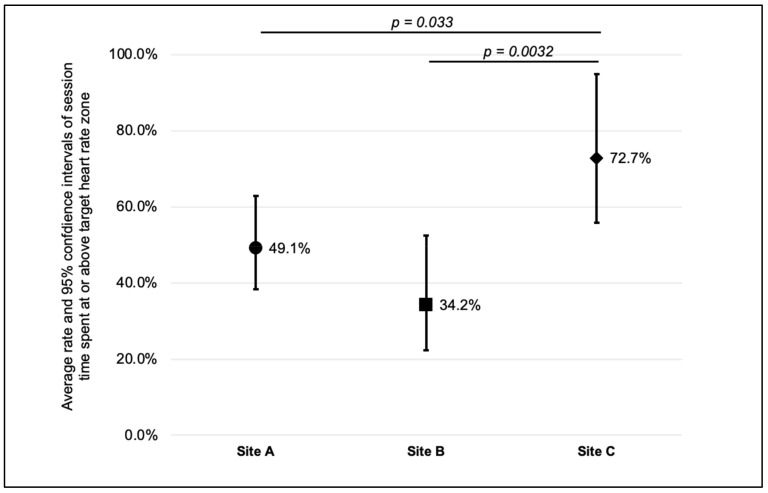
Average percentage and 95% confidence intervals of session time spent in or above participant target heart rate zone, by site.

Attendance at the sessions was high. On average, participants attended 88% of sessions and was similar across sites; at the session level, attendance ranged from 69% to 100%. Two participants withdrew from the study: one moved out of the area and the other lost interest and withdrew. Overall, girls spent 52.3% (95% CI: 39.3% to 65.3%) of each session engaged in MVPA; defined as the time in or above their individual target HR zone. Interestingly, the pattern of engagement also varied substantially between and within sites (Figure 1, *p* < 0.001). The pattern across the 12 weeks differed significantly by the site (*p* < 0.001); site A decreased, site B increased, and site C remained constant. As observed in Figure 2, the highest mean percentage of session time participants spent in or above their target HR zone was observed at Site C (71.6%, 95%CI: 52.5% to 90.7%) and the lowest in Site B (36.7%, 95%CI: 20.4% to 53.0%). MVPA engagement levels varied among participants; one girl averaged 4% of the dance session in her target HR zone and another averaged 94%.

Overall, we observed a non-significant mean increase of 73.4 feet in distance walked on the 6MWT from baseline to post-test for the 14 participants with data at both time points (*p* = 0.22, Table 2). There were similar increases in distance walked at each site, which ranged from an increase of 55.7 to 82.9 feet; however, none of these differences were statistically significant (Table 2).

Participants reported enjoying the dance sessions. On average, over the 10-week period that participant enjoyment was assessed (weekly for weeks 3 through 12), 83.7% (95% CI: 74.2% to 95.2%) of participants indicated they liked the type of dance, 80.3% (95% CI: 63.1% to 97.5%) liked the music played, and 85.0% (95% CI: 74.0% to 96.0%) liked dancing with other girls. There were only small differences in participants’ enjoyment across sites. With respect to perceived physical activity intensity, the majority (72.8%, 95% CI: 53.4% to 92.2%) reported that they worked “really hard” during sessions. Notably, Site A had the lowest prevalence of participants that worked “really hard” (48.4%, 95% CI: 20.9% to 76.9%) and 100% of Site B participants reported working “really hard” (Table 3).

Surveys with participants at week 12 reflected a high level of satisfaction with the program overall (Table 4). The assessment at week 6 found a similarly high and consistent level of satisfaction as the week 12 assessment. At both time points, 82.4% of the girls reported that they “liked” the dance program overall, and none reported disliking it. By the end of the program, most girls (76.5%) found the level of difficulty just right and none found it too hard. Almost all participants perceived improved fitness (88.2%), and almost all wished to continue dancing after the program ended (88.2%) and wanted to come back to the program (88.2%). Interestingly, most girls also indicated a preference for a girls-only dance program (87.5%) and for a program exclusively for those enrolled in special education (64.7%).

Parents of the participants in Site B (only) also reported their daughters had high levels of satisfaction with the program (Table 5). At the end of the intervention, 100% of those reporting agreed or strongly agreed that their daughter enjoyed the dance program, 100% agreed or strongly agreed that the program was taught at an appropriate level, and 90% thought dancing with one’s peers was important. All of those reporting indicated that their daughter learned new dance steps and routines. Fitness improvements were less consistently perceived: 67% of parents agreed or strongly agreed with the statement that their daughter’s fitness had improved over the course of the program, 83% reported that their daughter had shown new interest or motivation for participation in dance and that their daughter’s physical activity level had increased from before the program. All parents at Site B were interested in re-enrolling their daughter in the program.

The study protocol included directions for at-home practice with monitoring using a dance log and a Fitbit^®^ Charge HR™. On average, 54% of participants completed the Fitbit^®^ dance logs. There was substantial variation across the sites: 20% of logs were completed by participants at Site A and 40% at Site C. In contrast, more than 95% of logs were completed by participants at Site B. Table 6 summarizes the parent survey for Site B. Of the six parents who completed the post-intervention survey, three parents indicated that their child had practiced at home less than once per week; their data are tabulated in Table 6. Based on the parent report, two girls practiced 1–2 times per week: one girl danced every day. Barriers to the at-home practice varied and included practical considerations. The most frequently cited facilitating factor was having someone else dance with the participant. Parents varied regarding the difficulty of using the Fitbit^®^ Charge HR™ when their daughter danced at home.

## 4. Discussion

Our pilot dance intervention was successfully implemented in three distinct venues, reflecting a successful partnership among our research team, local community organizations, and a large public school. Overall, our results suggest that dance represents a promising approach to increasing time spent in MVPA in adolescent girls with ID and thereby could help girls with ID meet current physical activity recommendations. Girls enrolled in the study spent more than half of the 75-min session at an intensity level considered moderate to vigorous, based on heart rate monitoring. This intensity level was confirmed by participants; most participants reported they worked “really hard” during sessions. Engagement during the sessions varied substantially, based on time in or above their target heart rate zone. We did not observe increases in cardiorespiratory fitness as measured by the 6MWT, although a modest non-significant increase in distance walked between pre- and post- fitness testing was observed.

Our pilot effort adds to the small number of dance interventions for children, adolescents, and young adults with ID [54,55,56,57,58]. The five previously published dance interventions conducted with adolescents with ID examined outcomes that included balance, coordination, and locomotor skills; none measured MVPA by heart rate or cardiorespiratory fitness by 6MWT as we did in the present study. Only one previous investigation employed a randomized controlled trial study design, [54] and, like our study, the remaining four studies employed pre-post designs [55,56,57,58] and lacked a control group. Additionally, like ours, these studies were typically small, enrolling 10 to 36 participants. Prior work focused on younger children, ages 4 to 16.9 years. Participant diagnoses also varied, enrolling participants with Down syndrome [54,56] and autism spectrum disorder [58]. A trial of 36 Indian children aged 6–10 years with Down syndrome randomized to either traditional Indian dance or neuromuscular coordination exercises demonstrated that those who were randomized to dance experienced greater improvements in locomotor skills, but showed similar improvements in balance after the 6-week intervention period [54]. The four other smaller intervention studies with pre-post trial designs reported significant improvements in coordination, balance, vertical jump, flexibility, and sprint [55,56,57,58]. It is noteworthy that all published intervention studies in this population have demonstrated positive fitness outcomes, though mostly in skill-related fitness [54,55,56,57,58]. Our study adds to this research base in our positive, albeit variable, MVPA findings using heart rate monitoring.

We found very high levels of satisfaction with the program by participants overall. None of the previous dance intervention studies have reported participant satisfaction. Parents of participants in an adapted dance intervention for youth with Down syndrome reported high levels of satisfaction on a 10-point scale for almost all participants [56]. Interestingly, most participants in our study did not favor including boys in the program, nor did they favor including girls with typical development who were not receiving special education. There have been numerous calls to increase the capacity and accessibility of existing physical activity programs to meet the needs of youth with disabilities, rather than focusing efforts on developing specialized, separate programs that promote exclusion [59,60,61]. The preference for exclusive programming in our study may reflect past negative experiences in inclusive activities or their general overall satisfaction with the current program [62,63,64].

The at-home practice component was not well-received, as evidenced by both low reported compliance and barriers identified by parents who responded to the post-survey. Participants were encouraged to dance outside of the group sessions by the instructor and were verbally commended when they reported dancing at home. More deliberate strategies are clearly needed to stimulate this change in behavior, however. The videos may not have been sufficiently motivating to engage the girls, nor was wearing the Fitbit^®^ while dancing. With a growing array of dance-focused activities online, in apps, and streamed through social media (e.g., TikTok), getting adolescent girls with ID to dance outside of group programs may now be far easier.

Our study had several notable limitations in addition to its strengths. As a pilot, we focused on assessing the feasibility and acceptability of the intervention and did not include a control group. Despite having three implementation venues, our sample size was small. We did not collect data on other relevant fitness outcomes, such as dynamic or static balance or coordination. Notwithstanding these limitations, our study also had numerous strengths. Our design allowed us to demonstrate intervention feasibility in three distinct settings. Despite the multiplicity of venues, the intervention content, which featured two popular dance forms, was the same across all sites and delivered by the same instructor. In addition to gathering cardiorespiratory fitness data at multiple time points, satisfaction and preference information was obtained from the girls themselves, which represents an essential and overdue advance over prior studies [65]. We also were successful in partnering with community-based organizations, which may support program sustainability by building capacity for uptake in programming beyond a discreet research project. Finally, we were able to recruit a very diverse sample of adolescent girls with ID, who represent a woefully underrepresented and underserved population along several dimensions.

## 5. Conclusions

Our intervention was successful in that it resulted in participants engaging in dance at an MVPA intensity level for more than half of the 75-min dance sessions. Satisfaction with the dance program was high: the girls themselves expressed their enjoyment of the program and wanted it to continue. Together, this suggests that dance represents a viable approach to help adolescent girls with ID meet current physical activity guidelines. Further, partnering with community-level organizations holds promise for fostering inclusion in community and other settings (46).

Given the success of this pilot study for increasing engagement in physical activity, several directions for future study emerge. Establishing feasibility and preliminary efficacy in children with other developmental disabilities such as autism and among boys would increase the potential for including this program in school settings. A properly powered cluster-randomized controlled trial that includes an attention comparison group is needed to demonstrate efficacy. Additionally, to achieve an impact on cardiorespiratory fitness, more frequent training would be necessary; the at-home component of the intervention would also require additional development and implementation in a pilot context.

## Figures and Tables

**Table 1:** Characteristics of the study sample.

	All Sites (*n* = 18)	Site A (*n* = 6)	Site B (*n* = 7)	Site C (*n* = 5)
Age in years, mean (SD)	17.3 (2.7)	18.4 (2.5)	16.0 (2.8)	17.7 (2.3)
Race/ethnicity, *n* (%)				
Non-Hispanic White	8 (44.4)	3 (50.0)	4 (57.1)	1 (20.0)
Non-Hispanic Black	1 (5.6)	0 (0.0)	0 (0.0)	1 (20.0)
Hispanic	1 (5.6)	0 (0.0)	0 (0.0)	1 (20.0)
Asian	4 (22.2)	2 (33.3)	1 (14.3)	1 (20.0)
More than one race	4 (22.2)	1 (16.7)	2 (28.6)	1 (20.0)
Max parental education, *n* (%)				
Less than a bachelor’s degree	8 (44.4)	6 (100.0)	0 (0.0)	2 (40.0)
Bachelor’s degree or higher	10 (55.6)	0 (0.0)	7 (100.0)	3 (60.0)

**Table 2:** Six-minute walk test results pre- and post-intervention.

Distance in Meters	All Sites (*n* = 14) ^1^	Site A (*n* = 5)	Site B (*n* = 5)	Site C (*n* = 4)
Pre-Intervention, mean (SD)	469.7 (75.2)	464.9 (120.0)	472.7 (36.8)	475.0 (57/5)
Post-intervention, mean (SD)	492.5 (38.9)	486.7 (36.3)	497.7 (36.9)	492.8 (53.7)
Change between Pre- and Post-Intervention, mean (95% CI)	22.4 (−15.9 to 60.6)	24.9 (−38.8 to 87.6)	25.3 (−38.4 to 89.0)	17.0 (−17.0 to 88.2)
*p*-value	0.22	0.41	0.40	0.61

^1^ Three participants were missing data for the walk test (pre-intervention, *n* = 1; post-intervention, *n* = 2) and one participant was missing data for both pre- and post-intervention.

**Table 3:** Assessment of participants’ enjoyment of the dance program over the 12-week dance program.

	All Sites (*n* = 18) % (95% CI)	Site A (*n* = 6) % (95% CI)	Site B (*n* = 7) % (95% CI)	Site C (*n* = 5) % (95% CI)
Attendance	87.8 (84.2 to 91.6)	89.2 (80.9 to 94.1)	88.5 (81.4 to 93.1)	85.5 (81.1 to 88.9)
Liked dance style	83.7 (74.2 to 95.2)	79.3 (55.9 to 92.1)	86.6 (63.4 to 96.0)	84.0 (58.6 to 95.2)
Liked music played	80.3 (63.1 to 97.5)	74.7 (43.3 to 92.0)	83.8 (43.3 to 97.2)	87.5 (64.0 to 96.5)
Liked dancing with other girls	85.0 (74.0 to 96.0)	72.3 (51.8 to 86.4)	100.0 — ^1^	82.0 (62.1 to 92.7)
Worked really hard	72.8 (53.4 to 92.2)	48.4 (20.9 to 76.9)	100.0 — ^1^	71.2 (43.7 to 88.7)
Tried and liked the at-home videos	49.0 (31.3 to 66.6)	55.4 (36.5 to 72.8)	100.0 — ^1^	32.0 (12.7 to 60.3)

Satisfaction surveys were administered once per week starting with Week 3 of the intervention 12-week intervention. A total of ten surveys were administered. ^1^ 95% confidence intervals are not provided because all participants reported that they “liked” dancing with other girls, worked “really hard,” and tried and reported liking the at-home videos.

**Table 4:** Assessment of participant satisfaction with the dance intervention program post-intervention.

	Post-Intervention (*n* = 17) ^1^ *n* (%)
Overall satisfaction with the program	
Did not like it	0 (0.0)
It was okay	3 (17.7)
Liked it	14 (82.4)
How difficult were the dance steps	
Too hard	0 (0.0)
Too easy	4 (23.5)
Just right	13 (76.5)
How much work	
Didn’t work at all	0 (0.0)
Worked a little	4 (23.5)
Worked a lot	13 (76.5)
Learned new dance steps	
No	0 (0.0)
Yes	100.0
Got more fit	
No	2 (11.8)
Yes	15 (88.2)
Want to come back to program	
No	2 (11.8)
Yes	15 (88.2)
Want to keep dancing after program	
No	2 (11.8)
Yes	15 (88.2)
Include other girls not in special education	
Girls in special education only	11 (64.7)
Other girls	6 (35.3)
Want to include boys	
Include girls only	14 (87.5)
Include boys	2 (12.5)

^1^ One participant did not complete the post-intervention satisfaction survey.

**Table 5:** Parent satisfaction with the dance program at post-intervention at site B only.

	Post-Intervention (*n* = 6) ^1^ *n* (%)
Daughter enjoyed the program	
Neutral/Disagree/Strongly Disagree	0 (0.0)
Strongly Agree/Agree	6 (100.0)
Program was at an appropriate level for their daughter to understand	
Neutral/Disagree/Strongly Disagree	0 (0.0)
Strongly Agree/Agree	6 (100.0)
Dancing with peers was important for their daughter’s participation	
Neutral/Disagree/Strongly Disagree	0 (0.0)
Strongly Agree/Agree	6 (100.0)
Daughter’s fitness improved	
Neutral/Disagree/Strongly Disagree	2 (33.3)
Strongly Agree/Agree	4 (66.6)
Daughter learned new dance steps and routines	
Neutral/Disagree/Strongly Disagree	0 (0.0)
Strongly Agree/Agree	6 (100.0)
Daughter’s physical activity level increased from before the program	
Neutral/Disagree/Strongly Disagree	1 (16.7)
Strongly Agree/Agree	5 (83.3)
Daughter gave a good effort during classes and at home	
Neutral/Disagree/Strongly Disagree	1 (16.7)
Strongly Agree/Agree	5 (83.3)
Daughter has shown new interest/motivation in participating in dance	
No	1 (16.7)
Yes	5 (83.3)
Noted positive changes in their daughter attributed to the program	
No	0 (0.0)
Yes	6 (100.0)
Interested in re-enrolling your daughter in the program	
No	0 (0.0)
Yes	6 (100.0)

^1^ Six parents completed satisfaction surveys post-intervention.

**Table 6:** Parent perceptions of the at-home dance videos and Fitbit monitors post- intervention at site B only.

	Post-Intervention (*n* = 6) *n* (%)
Average number of times daughter practiced dance at home using the video	
<1 time/week	3 (50.0)
1 to 2 times/week	2 (33.3)
3 to 4 times/week	0 (0.0)
5 to 6 times/week	0 (0.0)
Everyday	1 (16.6)
Barriers for daughter’s participation in at-home dance	
Did not enjoy dancing at home	1 (16.7)
Difficult to follow along to the video	0 (0.0)
Did not have an appropriate space	0 (0.0)
Daughter’s schedule was too busy	1 (16.6)
Daughter was too tired	1 (16.6)
Did not have the time to remind/encourage daughter	1 (16.6)
Factors that helped daughter’s participation in at-home dance	
Daughter has always danced a lot at home	2 (33.3)
Daughter enjoyed the video	1 (16.7)
Scheduled day/time	1 (16.7)
Reminded/encouraged daughter	2 (33.3)
Someone else would dance with daughter/motivate her	0 (0.0)
Gave daughter a reward	6 (100.0)
Level of difficulty using Fitbit when daughter danced at-home ^1^	
Not difficult at all	2 (40.0)
A bit difficult	0 (0.0)
Difficult	3 (60.0)
Really difficult	0 (0.0)

^1^ One participant was missing data for this item (*n* = 5).

## Data Availability

The data presented in this study are available upon request from the corresponding author.
